# Prostaglandins and Their Receptors in Eosinophil Function and As Therapeutic Targets

**DOI:** 10.3389/fmed.2017.00104

**Published:** 2017-07-19

**Authors:** Miriam Peinhaupt, Eva M. Sturm, Akos Heinemann

**Affiliations:** ^1^Institute of Experimental and Clinical Pharmacology, Medical University of Graz, Graz, Austria

**Keywords:** allergy, inflammation, respiratory and gastrointestinal tract, bone marrow, chemotaxis, endothelium

## Abstract

Of the known prostanoid receptors, human eosinophils express the prostaglandin D_2_ (PGD_2_) receptors DP1 [also D-type prostanoid (DP)] and DP2 (also chemoattractant receptor homologous molecule, expressed on Th2 cells), the prostaglandin E_2_ receptors EP2 and EP4, and the prostacyclin (PGI_2_) receptor IP. Prostanoids can bind to either one or multiple receptors, characteristically have a short half-life *in vivo*, and are quickly degraded into metabolites with altered affinity and specificity for a given receptor subtype. Prostanoid receptors signal mainly through G proteins and naturally activate signal transduction pathways according to the G protein subtype that they preferentially interact with. This can lead to the activation of sometimes opposing signaling pathways. In addition, prostanoid signaling is often cell-type specific and also the combination of expressed receptors can influence the outcome of the prostanoid impulse. Accordingly, it is assumed that eosinophils and their (patho-)physiological functions are governed by a sensitive prostanoid signaling network. In this review, we specifically focus on the functions of PGD_2_, PGE_2_, and PGI_2_ and their receptors on eosinophils. We discuss their significance in allergic and non-allergic diseases and summarize potential targets for drug intervention.

## The Prostanoid—Eosinophil Axis in Allergic Diseases

Atopy is a genetically determined disorder, which results in characteristic inflammatory responses to *per se* innocuous antigens. Atopic diseases can manifest in different tissues as allergic rhinitis, conjunctivitis, bronchial asthma, dermatitis, or food allergies, and are associated with a major reduction in quality of life and life expectancy. In addition, some diseases, such as intrinsic asthma, aspirin sensitivity, nasal polyposis, adenoid hyperplasia, or chronic idiopathic urticaria, share several clinical and pathophysiological aspects of allergy, but with less clear ties to allergens. The basic concept of atopic reactions is grounded in an inadequate activation of immune cells by both specific and non-specific stimuli, with a shift toward the type-2 spectrum of inflammatory mediators, such as interleukin (IL)-4, -5, -9, and -13 (1). In allergen-specific IgE-mediated hypersensitivity reactions mast cells release preformed and newly synthesized mediators [histamine, leukotriene C_4_, prostaglandin (PG) D_2_, TNFα, and many others] (2). This is the pivotal step in the inflammatory cascade as it initiates the early phase of an allergic reaction. On the one hand, these mediators provoke symptoms such as sneezing, nasal congestion, rhinorrhea, wheezing, skin rash, etc., on the other hand, they trigger the infiltration of innate and adaptive immune cells, which favors the development of the late phase response that is characterized by symptoms such as bronchoconstriction, mucus hypersecretion, edema, pain, heat, and erythema.

Eosinophils are regarded as crucial effector cells in chronic allergic inflammation. Activated eosinophils release an array of cytotoxic and pro-inflammatory mediators promoting mucosal damage in chronic asthma and allergic inflammation. The tissue damage repeatedly initiates repair mechanisms that can lead to imbalance of epithelial-to-mesenchymal transition (3, 4). Consequently, eosinophils also play a role in airway remodeling and angiogenesis in chronically inflamed tissue, and hence contribute to the progression of the disease (5, 6). Consequently, eosinophil-deficient mice are protected against allergen-induced pulmonary inflammation and airway hyperresponsiveness (7, 8). The pathogenic role of eosinophils was eventually highlighted in a pivotal study showing that patients whose treatment is adjusted according to sputum eosinophil counts have significantly fewer severe asthma exacerbations than patients on standard management therapy (9). Therefore, eosinophils are currently considered a major therapeutic target in allergic diseases, such as conjunctivitis, rhinosinusitis, asthma, and atopic dermatitis, but they might also play pathogenic roles in several other diseases, such as eosinophilic esophagitis and gastroenteritis, pancreatitis, colitis ulcerosa, hypereosinophilic syndrome, renal disease, and cancer (10–19).

Importantly, the role of eosinophils in murine models of allergic airway inflammation is discussed controversially. IL-5 transgenic mice show pronounced eosinophilia and intrinsic airway hyperreactivity whereas the latter is abolished when CD4^+^ cells are depleted in these mice (20). However, it has also been observed that IL-5 transgenic mice are protected from airway hyperreactivity, and eosinophils isolated from BAL of OVA-challenged IL-5 transgenic mice do not release superoxides when activated with physiological stimuli (eotaxin, IL-5, PAF, or IgG) (21), which is in sharp contrast to human eosinophils. Therefore, the role of mouse vs. human eosinophils might differ in the pathophysiology of allergic diseases.

Human eosinophils express a distinct pattern of prostanoid receptors, comprising the receptors for PGD_2_, DP1 [also D-type prostanoid (DP)] (22) and DP2 [also chemoattractant receptor homologous molecule expressed on Th2 cells (CRTH2)] (23), the prostaglandin E_2_ receptors EP2 and EP4 (24), and the PGI_2_ (prostacyclin) receptor IP (25). When activated, these seven-transmembrane receptors couple to G proteins, which initiate further intracellular signaling events and are eventually eliciting a cellular response. Depending on the G protein subtypes involved, this can lead to the activation of opposing signaling pathways (26–29). For instance, the DP2 receptor couples to Gα_i_ and Gα_q_ causing eosinophil shape change and migration, while the IP receptor inhibits these eosinophil responses, likely through Gα_s_. In the mouse, eosinophils express DP1 and DP2 (30). EP2 is expressed on murine eosinophils since the EP2 agonist butaprost inhibits eosinophil trafficking, and in OVA-sensitized mice, the infiltrating leukocytes after allergen challenge were immunohistologically stained EP2 positive (31). The expression of EP1, EP3, EP4, and IP remains elusive; however, IP-deficient OVA-sesitized mice show less eosinophils in the brochoalveolar lavage and airway inflammation after allergen challenge as compared to wild type mice (32, 33).

## Prostaglandin D_2_ (PGD_2_)

Prostaglandin D_2_ is the principal ligand for two receptors, DP1 and DP2 (34), of which both are expressed on the surface of eosinophils (35). At micromolar concentrations, PGD_2_ is also an agonist of the thromboxane receptor, TP, which mediates the direct bronchoconstrictor effect of PGD_2_ (36). Moreover, a major metabolite of PGD_2_, 15-deoxy-Δ^12,14^-PGJ_2_ is a potent agonist of peroxisome proliferator-activated receptor (PPAR)-γ, which is also expressed by eosinophils (37). PGD_2_ had been known to stimulate eosinophil locomotion for some time (38, 39), but it was only in 2001 that the DP2 receptor was found to mediate this effect (22, 40, 41). Also, DP2 activation by PGD_2_ or DP2-selective ligands triggers Ca^2+^ flux, CD11b upregulation, respiratory burst, and release of eosinophil cationic protein (22, 40–42). Eosinophil responses to DP2 activation seem to depend on Gα_q_ proteins, exemplified by the lack of effect of pertussis toxin on PGD_2_-induced eosinophil shape change, which—however—is abrogated by phospholipase C inhibition (43). However, PGD_2_-induced chemotaxis was abrogated by pretreatment of eosinophils with pertussis toxin (unpublished observation). In addition to directly stimulating eosinophil migration, we also observed that PGD_2_ is capable of priming eosinophils for other chemoattractants like eotaxin, 5-oxo-6,8,11,14-eicosatetraenoic acid (5-oxo-ETE), or complement factor C5a, an effect that is likewise mediated by the DP2 receptor (42, 44). Conversely, eosinophil migration toward PGD_2_ is impaired by eotaxin or 5-oxo-ETE in a pathway depending on phosphoinositide 3-kinase as well as p38 mitogen-activated protein kinase (44). The subcellular signaling cascades that mediate the priming effect of PGD_2_ are not yet understood, while the priming effect of the PGD_2_ metabolite 15-deoxy-Δ^12,14^-PGJ_2_ seems to involve PPAR-γ (45). Thus, it appears that a hierarchy exists among eosinophil chemoattractants: PGD_2_ might be regarded as an initial chemoattractant, since its potency is sustained also in whole blood and primes eosinophils for other chemoattractants; however, eotaxin seems to be an end-point chemoattractant, as it has reduced efficacy in blood as compared to isolated eosinophils, and effectively downmodulates eosinophil migration toward other chemoattractants (44).

Besides PGD_2_, DP2 is also activated by the PGD_2_ metabolites 13,14-dihydro-15-keto- (DK-) PGD_2_, PGJ_2_, Δ^12-^PGJ_2_ and 15-deoxy-Δ^12,14^-PGJ_2_ (42, 46, 47). Considering that PGD_2_ is as short-lived molecule and rapidly degraded into metabolites (48), it is interesting that the PGD_2_ actions on eosinophils are maintained through metabolites binding to DP2. Moreover, one of the major metabolites of the thromboxane pathway, 11-dehydro-TXB_2_, and even the common precursor of all prostanoids, PGH_2_, are also potent DP2 agonists (49, 50). Similarly, PGF2α has been found to activate eosinophils through DP2 (51).

In human disease, DP2 on peripheral blood eosinophils is upregulated in allergic dermatitis and rhinitis patients (52, 53), but it is diminished in active ulcerative colitis (26).

Although PGD_2_ binds to DP1 with similar affinity as to DP2 (34), the exact function of this receptor in immune cells has not been fully elucidated yet, and both pro- and anti-inflammatory effects have been reported (29). For instance, DP1 mediates the PGD_2_-induced expression of the airway mucin MUC5B in human nasal epithelial cells (54) and stimulates mucus production *in vitro* (55) but inhibits the functions of platelet, neutrophils, basophils, and dendritic cells (56–62). Unlike DP2, which is preferentially expressed on immune cells, such as eosinophils, basophils, macrophages, mast cells, a subset of Th2 lymphocytes and group 2 innate lymphoid cells (23, 40, 63–66), DP1 is more widely expressed, including the vasculature, the central nervous system, the retina, and the lungs (55, 67–69).

DP1-deficient mice were shown to be protected from development of allergic lung inflammation in terms of airway hyperresponsiveness, reduced numbers of BAL eosinophils, and BAL levels of IL-4, IL-5, and IL-13 (70). In contrast, intratracheal administration of DP1 agonist BW245c protected mice from airway hyperresponsiveness and lung eosinophilia in a OVA models of experimental asthma, thereby counteracting DP2-mediated proinflammatory responses (30, 71). DP1 activation has also been linked to inhibition of dendritic cell function (60) and to reduce inflammation in an IL-10-dependent mechanism (71). DP1, but not DP2, expression in lung tissue (mRNA) is upregulated upon OVA challenge (72). More recently, in guinea pigs, PGD_2_ aerosols were shown to induce the activation of sensory nerves and cough *via* DP1 receptor activation. Interestingly, DK-PGD_2_ modulated the sensory nerve activity by inhibiting the response to capsaicin (73).

**Figure 1:**
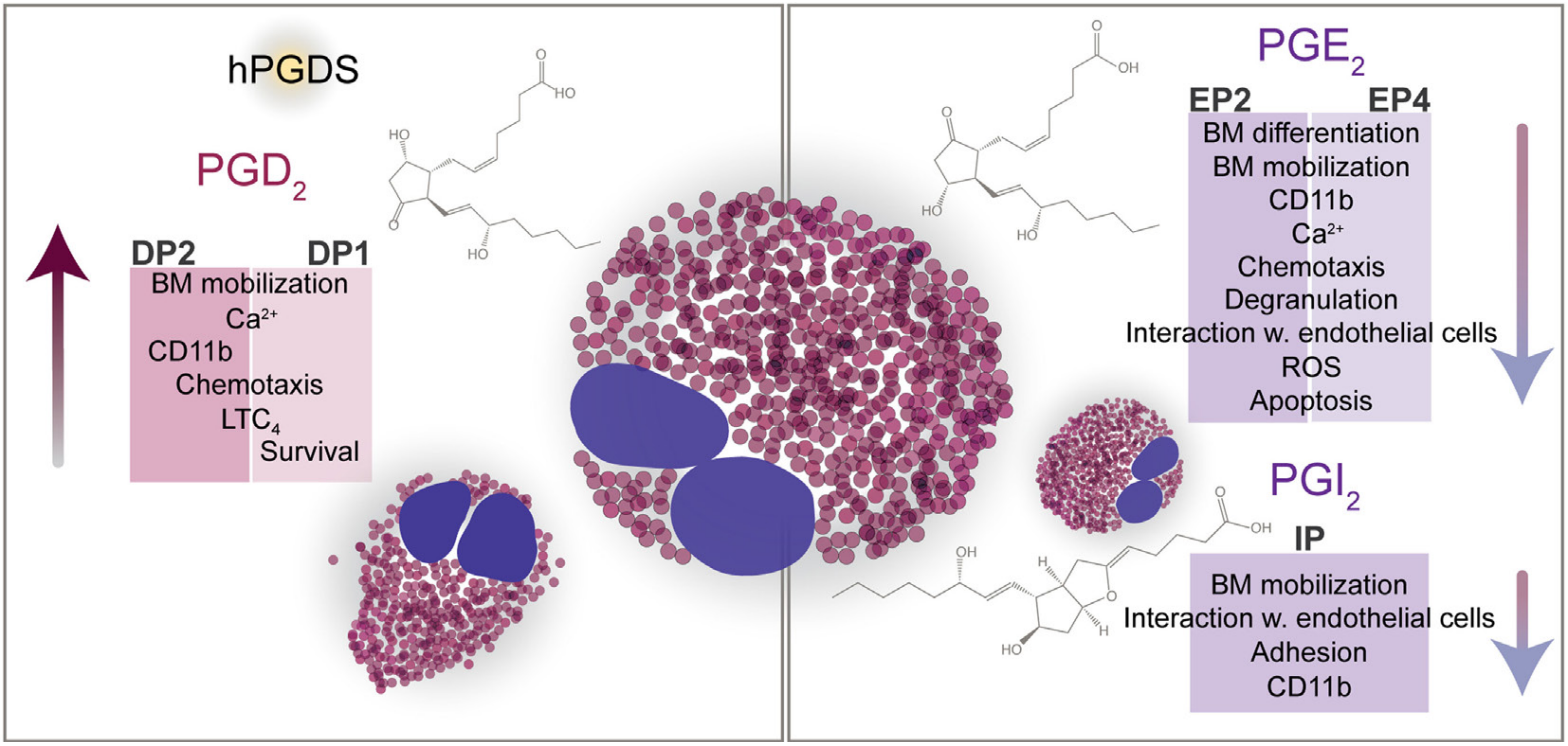
PGD_2_, PGE_2_, and PGI_2_ direct the functions of eosinophils. Eosinophils express receptors for PGD_2_ (DP1, DP2), PGE_2_ (EP2, EP4), and PGI_2_ (IP). *Via* DP2, PGD_2_ attracts eosinophils to the site of inflammation, enhances eosinophil mobilization from the bone marrow, and upregulates CD11b expression. In line with the chemotactic response, PGD_2_-mediated activation of eosinophils results in increased size and altered cell shape. DP1 and DP2 cooperatively regulate the synthesis of LTC_4_. DP1 has been shown to enhance the DP2-mediated Ca^2+^ response and to prolong the survival of eosinophils *in vitro*. Counteracting pro-inflammatory mechanisms PGE_2_ and PGI_2_ suppress the activation of eosinophils and hence dampen pro-inflammatory signals. Despite the negative regulation of eosinophil effector and chemotactic functions by PGE_2_ and PGI_2_, PGE_2_ was shown to decrease eosinophil apoptosis *in vitro*.

In eosinophils, the DP1 receptor transmits antiapoptotic signals by PGD_2_ (22), but has been found to limit DP2-mediated CD11b upregulation (41). At micromolar concentrations, however, PGD_2_ and 15-deoxy-Δ^12,14^-PGJ_2_ drive eosinophils into apoptosis in a nuclear factor κB-dependent manner (74). Regarding other eosinophil responses, there is growing literature reporting cooperative signaling of DP1 and DP2 receptors. In guinea pigs, both DP1 and DP2 activation can stimulate the mobilization of eosinophils from the bone marrow (75). Moreover, DP1-dependent eosinophil responses such as migration and production of reactive oxygen species are—to some extent—co-mediated by DP1 (75, 76). On the molecular level, we have shown that DP1 activation is substantially involved in DP2-triggered Ca^2+^ signaling in a heterologous expression system and in human peripheral blood eosinophils and, therefore, might be an important regulator of DP2-mediated pro-inflammatory signaling (35). Cooperative signaling of the two receptors also converges in the PGD_2_-induced synthesis of leukotriene C4 synthesis in eosinophils. Only a simultaneous activation of DP1 and DP2 led to a sufficient response while the activation of either one or the other receptor did not equal the full PGD_2_ response (77). This finding does not only substantiate the significance of PGD_2_ in stimulating the synthesis of LTC_4_ but also highlights the cooperative function of the two PGD_2_ receptors (Figure 1).

## Targeting PGD_2_ Signaling in Eosinophilic Diseases

### DP2 Receptor Antagonists

Blood and tissue eosinophilia is a key feature of allergy and asthma. It correlates with the severity of the disease on the one hand, and levels of PGD_2_ on the other hand (78). Exogenously applied PGD_2_ and DP2 agonists provoke peripheral blood eosinophilia and infiltration of eosinophils into the conjunctiva, lung, nose, and skin in animal models (30, 38, 79–82), whereas pharmacological blockade of DP2 can ameliorate models of atopic dermatitis, asthma, rhinitis, and conjunctivitis (83–88). Interestingly, DP2-deficient mice develop a normal chronic allergic inflammatory response to allergen challenge after sensitization and challenge, while the acute inflammatory response and eosinophil infiltration in the skin are abrogated (89).

The effects of the DP2 antagonist timapiprant (OC-459) was studied in a large patient cohort (*n* = 482) of mild-to-moderate persistent asthma. In this randomized, double-blind placebo-controlled study, the DP2 antagonist was given over 12 weeks with overall beneficial effects on lung function. A *post hoc* analysis revealed that the greatest improvement of lung function by timapiprant was observed in patients with active eosinophilia (≥250/μl peripheral blood) and—even more pronounced—in younger patients (90). This applies also for the humanized murine IL-5 antibody mepolizumab, which is most effective and only given in asthma patients with severe eosinophilic airway inflammation (91). In eosinophil esophagitis, timapiprant significantly reduced the esophageal eosinophil load and induced some clinical improvement (92). Timapiprant also successfully reduced nasal and ocular symptoms in allergic subjects exposed to grass pollen (93).

Fevipiprant (QAW039) is another DP2 antagonist, but as compared to timapiprant, it has slower dissociating properties and is, therefore, a candidate compound with potentially improved efficacy (94). In 170 patients with uncontrolled asthma, however, fevipiprant administered once daily did not meet the overall expected primary clinical end point (increase in FEV_1_), but led to an improvement of clinical symptoms in a sub-cohort with severe asthma (FEV_1_ < 70%), leading to a significant improvement in FEV_1_ and the asthma control questionnaire score, in addition to being well tolerated by the patients (95). It has to be considered, however, that *post hoc* analyses like these performed with fevipirant and timapiprant (90) need to be interpreted with caution. Importantly, fevipirant reduced eosinophilic airway inflammation in a separate, small trial comprising 61 patients with persistent moderate-to-severe asthma, uncontrolled by inhaled corticosteroids and elevated sputum eosinophil counts (96).

Several other DP2 antagonists have been subject to clinical trials in asthma or even COPD, but showed little efficacy and are discussed elsewhere (97).

### DP1 Receptor Antagonists

Based on *in situ* hybridization and immunohistochemistry, DP1 mRNA and DP2 protein expression were detectable in eosinophils in nasal polyp tissue of allergic rhinitis patients; in contrast, only DP1 but not DP2 was observed in nasal tissue of healthy subjects (67).

A pivotal study using DP1-knockout mice suggested that DP1 plays an important role in the OVA-induced asthma model. DP1-deficient mice not only showed markedly reduced eosinophils in BAL fluid but also did not develop airway hyperresponsiveness (70). In a rat model of OVA-induced pulmonary inflammation, DP1 expression was upregulated in the lungs while bronchial hyperresponsiveness and immune cell infiltration was diminished by the DP1 antagonist S-5751 (98). In an OVA-induced allergic rhinitis model in guinea pigs, S-5751 inhibited late phase responses such as infiltration of eosinophils and mucosal plasma exudation (99). A newly developed DP1 antagonist (S-555739, asapiprant) showed improved affinity and bioavailability, and reversed antigen- and PGD_2_-induced nasal congestion and airway hyperresponsiveness in guinea pigs and sheep, respectively, along with significantly decreased eosinophils and other inflammatory cells in nasal lavage fluid (100). A phase II clinical trial in the USA (NCT01651871) and a phase III clinical trial (JapicCTI-132046) in Japan are underway testing asapiprant in seasonal allergic rhinitis. The results are yet to be announced. Previously, another DP1 antagonist, laropiprant (MK-0524), was shown to prevent nasal congestion induced by PGD_2_ in healthy subjects (101) but failed in phase II trials in allergic rhinitis and asthma (102). Similarly, the dual DP1/DP2 antagonist vidupiprant (AMG 853) provided no benefit as an add-on to inhaled corticosteroid therapy in moderate-to-severe asthma (103).

### Inhibition of PGD_2_ Synthases—HPGDS and Lipocaline Prostaglandin D_2_ Synthase (LPGDS)

In mammals, two isoforms of PGD_2_ synthases are expressed: the lipocaline type (LPGDS), which is highly abundant in the central nervous system and the hematopoietic type (HPGDS), which is mainly expressed in mast cells, but also can be found in macrophages and Th2 lymphocytes (Table 1). Additionally, resident eosinophils themselves might be a late source of PGD_2_ at the site of allergic inflammation acting in an autocrine manner to attract and activate further eosinophils (104, 105). An interesting novel link between PGD_2_ and eosinophils is the recent discovery of pro-eosinophilic, so-called pathogenic effector (pe)Th2 lymphocytes, which highly express IL-5 and IL-13, and can be found at elevated levels in eosinophilic patients suffering from atopic dermatitis and eosinophilic gastrointestinal disease. These cells express not only DP2 but also HPGDS (106).

**Table 1:** PGD_2_ release and expression of hematopoietic prostaglandin D_2_ synthase (HPGDS) and lipocaline prostaglandin D_2_ synthase (LPGDS) in human cells.

Cell type	HPGDS	LPGDS	PGD_2_ release
Astrocytes	Mohri et al. (68)		
Basophils	Tanaka et al. (107); Dahlin et al. (108)		
Dendritic cells	Shimura et al. (109)		Shimura et al. (109)
Endothelial cells		Taba et al. (110)	Taba et al. (110), Camacho et al. (111)
Eosinophils	Luna-Gomes et al. (112)		Luna-Gomes et al. (112)
Epithelial cells (choroid plexus)		Blödorn et al. (113)	
Bronchial epithelial cells (HBEC)			Jakiela et al. (114) (mass spectrometry)
ILC2	Björklund et al. (115)		
Keratinocytes			Kanda et al. (116)
Langerhans cells (epidermal)	Shimura et al. (109)		
Macrophages	Jandl et al. (65)		Tajima et al. (117)
Mast cell progenitors	Dahlin et al. (108)		
Mast cells	Nantel et al. (67)		Schleimer et al. (118); Lewis et al. (119)
Megakaryoblastic cells (CKM, Dami cells)	Mahmed et al. (120); Suzuki et al. (121)		
Microglia	Mohri et al. (68)		
Myocardial cells		Eguchi et al. (122)	
Oligodendrocytes		Mohri et al. (68); Kagitani-Shimono et al. (123)	
Osteoarthritic chondrocytes		Zayed et al. (124)	Zayed et al. (124)
Smooth muscle cells (arteriosclerotic plaques)		Eguchi et al. (122)	
Th2 subsets	Mitson-Salazar et al. (106); Tanaka et al. (107); Wang et al. (125); Nagata et al. (126)		

Both PGD synthases are regarded as promising drug targets in a variety of diseases, such as allergic inflammation, mastocytosis, asthma and chronic obstructive pulmonary disease, metabolic disorders, muscular dystrophy, Alzheimer’s disease, or spinal cord injury (127), stimulating the development of several selective inhibitors (128–136). Transgenic mice overexpressing LPGDS show exaggerated eosinophilic pulmonary inflammation (72), which was reversed by AT-56, a LPGDS inhibitor (129). In contrast, eosinophil numbers in OVA-induced pulmonary inflammation are not significantly increased in transgenic mice overexpressing HPGDS, but the HPGDS inhibitor HQL-79 abrogated eosinophilic pulmonary inflammation in OVA-challenged mice (128). HPGDS in healthy nasal mucosa is expressed only in mast cells, but in allergic rhinitis and nasal polyps also in infiltrating inflammatory cells including eosinophils (67, 137). In a guinea-pig model of allergic inflammation, the HPGDS inhibitor TAS-204 prevented OVA-induced nasal obstruction and eosinophil infiltration (132).

### Activation of PPAR-γ

In an OVA-induced allergic model, 15-deoxy-Δ^12,14^-PGJ_2_ and the PPAR-γ agonist rosiglitazone abrogated peritoneal accumulation of eosinophils and eosinophil proliferation in bone marrow (138). Similarly, several studies have shown that synthetic PPAR-γ agonists are beneficial in mouse models of allergic pulmonary inflammation and rhinitis (139, 140). Pioglitazone was tested in patients with mild asthma but did not reproduce the results from animal studies (141).

## Prostaglandin E_2_

Infiltration of eosinophils along with other proinflammatory parameters in OVA-induced asthma model was found to be markedly enhanced in COX-1 and COX-2 knockout mice (142) and after pharmacological blockade of these enzymes (143). Conversely, inhaled PGE_2_ reduced airway inflammation, hyperresponsiveness, and eosinophil counts in BAL fluid of asthmatic patients (144). These findings suggested a possible inhibitory effect of PGs on eosinophils.

In airways, PGE_2_ is released by epithelial-, endothelial-, and smooth muscle cells, macrophages, and fibroblasts, and potently counteracts the pro-inflammatory actions of PGD_2_. PGE_2_ has bronchodilator functions and reduces airway hyperresponsiveness *via* activation of EP2 receptors (145). Recently, we found that PGE_2_ promotes the endothelial barrier by EP4 receptors expressed on the endothelium and protects against thrombin-induced junctional disruption (146).

Early studies indicated that PGE_2_ inhibits the release of eosinophil cationic protein (39) and homotypic aggregation of eosinophils (147) that is mediated by the β2-integrin CD18 (148). Of the known PGE_2_ receptors (EP1, EP2, EP3, and EP4), eosinophils express mRNA for EP2 and EP4 (24). Accordingly, we found both EP2 and EP4 protein in eosinophils using flow cytometry and Western blot, respectively (27, 31). By directly addressing the significance of PGE_2_ in eosinophil function, we could show that PGE_2_ acts to suppress eosinophil responses such as chemotaxis and degranulation, which seemed to be mediated by both EP2 and EP4 receptors (27, 31). On the subcellular level, EP4 receptor activation resulted in blockade of intracellular Ca^2+^ release, cytoskeletal reorganization, and production of reactive oxygen species (27). EP4 agonist treatment inhibited CD11b upregulation, activation, and clustering of β2 integrins, and L-selectin shedding of eosinophils, which were all abolished using an EP4 antagonist (149). We could delineate the underlying signaling pathways to involve phosphoinositide 3-kinase, phosphoinositide-dependent kinase 1, and protein kinase C but not the cyclic AMP/protein kinase A pathway (27, 150). Likewise, the PGE_2_—EP4 axis acted inhibitory on the interaction of eosinophils with endothelial cells, including adhesion and transmigration (149). In contrast, mobilization of eosinophils from guinea pig bone marrow was mediated by the EP2 receptor (31). Previously, *in vitro* eosinophilopoiesis stimulated by IL-5 was also observed to be under negative control of PGE_2_ in normal and OVA-sensitized mice by selectively inducing apoptosis in developing eosinophils (151, 152). Unexpectedly, PGE_2_ has been found to be antiapoptotic for peripheral blood eosinophils (153, 154), which might be linked to elevated PGE_2_ levels in airways of asthmatic patients (155), and even more in non-asthmatic eosinophilic bronchitis (156). Another study, however, found an inverse relationship between sputum eosinophil counts and PGE_2_ levels (157). Nevertheless, activation of the EP2 receptor inhibited the allergen-induced increase of eosinophils in the bronchoalveolar lavage fluid of OVA-sensitized mice (31).

Hence, the activation of EP2/EP4 receptors can be protective against the accumulation and activation of eosinophils in the affected tissue, and is therefore considered as a potential treatment strategy in allergy (Figure 1).

## Prostaglandin I_2_

Parts of the immune-suppressive effects of PGE_2_ are shared by PGI_2_ (prostacyclin). In contrast to EP2/EP4 signaling, the activation of PGI_2_ receptors (IP) is mediated by intracellular cAMP, thereby inhibiting eosinophil functions. PGI_2_ and the stable PGI_2_ mimetic iloprost negatively regulate the trafficking of guinea pig bone marrow eosinophils *via* IP receptor activation (158). In experimental asthma in mice, iloprost attenuates dendritic cell function and the concomitant allergen-specific Th2 response and inhibits eosinophilia in lung tissue (159). After repeated allergen challenge, endogenous PGI_2_ abrogates airway remodeling (32).

In an *in vitro* study using human eosinophils and endothelial cells, we found that endothelium-derived PGI_2_ is an important modulator of eosinophil–endothelial interaction and might have a bearing on eosinophil accumulation at sites of allergic reaction. Moreover, PGI_2_ promotes the barrier function of lung endothelial cells and limits eosinophil adhesion and transendothelial migration (25). Our data might hence explain previous findings that deletion of IP receptors in mice augments the eosinophilic infiltrate in allergic responses of the lung and skin and enhances airway remodeling (32, 33).

## The Prostanoid—Eosinophil Axis in Non-Allergic Diseases

### Aspirin-Exacerbated Respiratory Disease (AERD)

Also referred to as aspirin intolerance or Samter’s triad, AERD is a chronic inflammatory state of the airways resulting in rhinosinusitis, nasal polyps, and asthma. In some patients, these symptoms are accompanied by skin rash such as urticaria or angioedema, while in others the skin manifestations are prevailing. These symptoms are aggravated after intake of aspirin (acetylsalicylic acid) or any other non-selective COX inhibitor, occasionally culminating in massive anaphylactoid reactions or even death. In contrast, selective COX-2 inhibitors are mostly tolerated. A comprehensive overview on clinical presentations and pathobiologic mechanisms is provided elsewhere (160–162). In brief, an imbalance of anti-inflammatory PGE_2_ and proinflammatory LTC_4_ exists in these patients at baseline, which is further enhanced after intake of COX inhibitors, which alludes into activation of mast cells, eosinophils, and several other immune cells. In addition to mast cells, LTC_4_ biosynthesis in eosinophils is upregulated in AERD patients. Similarly, both cell types express more HPGDS and release excessive levels of PGD_2_ in this condition (163). Urinary levels of a stable PGD_2_ metabolite were found to be twofold higher in patients with AERD relative to those in control subjects and—most remarkably—increased further upon aspirin exposure. This correlated with reductions in blood eosinophil counts and lung function, and clinical symptoms such as nasal congestion (164). Aspirin-induced secretion of PGD_2_ was abrogated after successful aspirin desensitization therapy (165). Aspirin by itself was found to activate blood eosinophils in terms of Ca^2+^ flux, degranulation, and CD11b upregulation, the latter being more pronounced in AERD patients (166, 167). These effects were reversed by PGE_2_. We observed that the expression of the EP4 receptor in blood eosinophils tended to be reduced in AERD patients, and inhibition of eosinophil chemotaxis by PGE_2_ or an EP4 agonist was less pronounced in AERD patients as compared to healthy controls (168). Single nucleotide polymorphisms of the *ptger2* and *ptger4* were detected in aspirin-intolerant Korean patients, predicting lower EP2 and EP4 receptor expression levels (169, 170). A single nucleotide polymorphism in the DP2 gene *crth2* was also observed to correlate with increased levels of the eosinophil chemoattractant, eotaxin-2 in Korean AERD patients (171). Similarly, the prevalence of a *crth2* single nucleotide polymorphism was found to be increased in a female Japanese AERD patient cohort (172). These findings suggest that targeting PGE_2_ and PGD_2_ receptors might provide potential novel treatment options for AERD. Whether these genetic alterations specifically contribute to AERDS pathophysiology is still unclear, as similar finding have also been made for allergic disease and asthma (173).

### Miscellaneous

Eosinophil infiltration into tumor-surrounding areas is observed in various types of cancer (174). The presence of tumor-associated tissue eosinophils (TATEs) seems to beneficially influence the prognosis of oral squamous cell carcinoma and other types of cancer. Davoine et al. have shown that eosinophil lysates inhibit the growth of the oral squamous carcinoma cells line (SCC-9) *in vitro* and correlates with the amount of released eosinophil peroxidase. Inhibition of HPGDS by HQL-79 in oral squamous cell carcinoma abrogated the migration of eosinophils toward the tumor cells. These results suggest an antitumor activity of PGD_2_ *via* the activation of release of eosinophil peroxidase from, or by cytolysis of, eosinophils (175). By using HPGDS-deficient mice, Murata et al. have shown that mast cell-derived PGD_2_ is an antiangiogenic factor in lung carcinoma (176). Therefore, stimulating the HPGDS/PGD_2_ axis could be a beneficial strategy in cancer, with TATEs serving as an additional biomarker.

Eosinophils have been shown to play a significant role in inflammatory bowel disease, ulcerative colitis, and Crohn’s disease (13, 177, 178). We have shown in experimental Crohn’s disease that eosinophils contribute to intestinal inflammation *via* activation of DP2. Timapiprant inhibited the recruitment of eosinophils into the colon, reduced intestinal inflammation, and decreases cytokine levels (TNFα, IL-1β, IL-6) in mice. In Crohn’s patients, PGD_2_ and Δ^12^-PGJ_2_ levels were increased as compared to control individuals (179). In a subsequent study, increased expression of LPGDS in myenteric and submucosal neurons, and enhanced PGD_2_ release, was observed in tissue samples from colon of patients with active Crohn’s disease (180). In ulcerative colitis, we observed opposing effects of DP1 and DP2 as blockade of DP2 improved, whereas a DP1 antagonist worsened, inflammation in a mouse model of colitis (26). In ulcerative colitis patients, DP2 expression was downregulated on peripheral blood eosinophils, while DP1 was upregulated, and both findings correlated with disease activity. Biopsies of colitis patients revealed an increase of DP2-positive cells in the colonic mucosa and high DP2 protein content. Both PGD_2_ and PGE_2_ levels were elevated in serum of colitis patients (26). Eosinophils and macrophages were suggested to be the main source of PGE_2_ in colitis (181). Current literature suggests that, like in allergy, PGE_2_ through its EP4 receptor opposes the pro-inflammatory action of PGD_2_ in inflammatory bowel disease and plays a protective role in mouse models of colitis (182–184). In contrast, a large body of evidence supports EP4 receptors to predominantly mediate the overall pro-tumorigenic action of PGE_2_ (185). Whether inhibition of eosinophil function is involved in the anti-inflammatory and pro-tumorigenic roles of the EP4 receptor in the gut has not been investigated yet.

## Concluding Remarks

Accumulating data suggest that the DP2 receptor is an important activator of eosinophils, as it does not only respond to its cognate ligand, PGD_2_, but also to most of its metabolites, and even unrelated prostanoid species. PGD_2_ is generated by a large variety of immune cells under different conditions. Among other leukocytes, eosinophils are probably the most important DP2-bearing cells. Thus, it is believed that DP2, and to some extent also DP1, crucially contribute to various pathologies that involve eosinophils, and provide novel therapeutic approaches to conditions such as asthma, allergic rhinitis, conjunctivitis, esophagitis and skin disease, nasal polyposis, aspirin-intolerance, Crohn’s disease, and certain types of cancer. In contrast, PGE_2_ transmits inhibitory signals onto eosinophils through EP2 and EP4 receptors, and is thus a natural antipode to its isomer, PGD_2_. For instance, HPGDS expression is enhanced, while microsomal PGE_2_ synthase is decreased in chronic rhinosinusitis that results in eosinophilic inflammation favoring polyp formation (186). In asthma patients, a decrease of PGE_2_ as compared to other prostanoids including PGD_2_ correlates with airway obstruction (187). Similar findings are typical for AERD. An imbalance of PGD_2_/PGE_2_ secretion might hence potentially underlie and/or sustain the abovementioned, eosinophilic pathologies, and might constitute novel therapeutic targets.

## Author Contributions

MP, ES, and AH wrote and edited the review.

## Conflict of Interest Statement

MP and ES declare that the research was conducted in the absence of any commercial or financial relationships that could be construed as a potential conflict of interest. AH received consulting fees from AstraZeneca and Bayer.
